# Tailgut Cyst—Gynecologist’s Pitfall: Literature Review and Case Report

**DOI:** 10.3390/diagnostics15010108

**Published:** 2025-01-04

**Authors:** Andrei Mihai Malutan, Viorela-Elena Suciu, Florin Laurentiu Ignat, Doru Diculescu, Razvan Ciortea, Emil-Claudiu Boțan, Carmen Elena Bucuri, Maria Patricia Roman, Ionel Nati, Cristina Ormindean, Dan Mihu

**Affiliations:** 1 2nd Department of Obstetrics and Gynecology, ‘Iuliu Hatieganu’ University of Medicine and Pharmacy, 400006 Cluj-Napoca, Romania; malutan.andrei@gmail.com (A.M.M.);; 2 “Ion Chiricuta” Oncoloy Institute, 34-36 Republicii, 400015 Cluj-Napoca, Romania; 3 Department of Pathology, Emergency County Hospital, 3-5 Clinicilor, 400006 Cluj-Napoca, Romania; 4 Clinical Department of Surgery, “Constantin Papilian” Emergency Clinical Military Hospital, 22 G-ral Traian Mosoiu, 400132 Cluj-Napoca, Romania

**Keywords:** tailgut cyst, retrorectal space, retrorectal hamartomas, chronic pelvic pain

## Abstract

**Background and Clinical Significance:** Retrorectal cystic hamartomas (“Tailgut cysts”) are rare developmental cysts that appear in the retrorectal space, arising from aberrant remnants of the post-anal primitive gut in case of an incomplete embryogenetic involution. **Case Presentation:** We present the case of a 30-year-old woman with a history of chronic lower abdominal pain. Other digestive symptoms, like rectal fullness, constipation, pain on defecation, rectal bleeding or genitourinary obstruction symptoms, were not associated. During a period of 3 years, she underwent several surgical procedures for ovarian cysts, without relieving the symptomatology. A computed tomography (CT) scan showed a presacral tumor with a right pararectal development. A surgical resection of the lesion using an anterior approach was performed, with the final pathological diagnosis of a retrorectal cystic hamartoma (“tailgut cyst”). **Conclusions:** This case underlines the fact that retrorectal masses can be challenging to diagnose, and tailgut cysts must be taken into consideration in cases of perirectal tumors in patients with a history of multiple failed procedures and surgeries.

## 1. Introduction

Retrorectal cystic hamartomas, commonly referred to as “tailgut cysts”, are rare developmental cysts that occur in the retrorectal space. These lesions are believed to originate from aberrant remnants of the post-anal primitive gut due to incomplete embryological regression [1,2]. Developmental cysts are classified based on their embryonic origin into categories such as epidermoid cysts, dermoid cysts, tailgut cysts, and rectal duplication cysts (also known as enteric or enterogenous cysts, or enterocystomas) [3,4].

The incidence of tailgut cysts is estimated to be 1 in 40,000 individuals. While these cysts can manifest across all age groups, they are most frequently observed in middle-aged women [5,6]. Clinical symptoms are generally nonspecific, with some patients remaining asymptomatic, contributing to diagnostic delays compounded by limited clinician familiarity with this condition. Magnetic resonance imaging (MRI) is the primary diagnostic tool for suspected tailgut cysts, whereas the utility of biopsies remains controversial [7,8,9].

The standard treatment involves complete surgical excision with negative margins [10,11,12]. Notably, Hjermstad and Helwig conducted the largest series on tailgut cysts to date, reporting 53 cases over a 35-year period in their 1988 publication [1,13].

We aim to present a rare case of retrorectal cystic hamartoma, diagnosed after multiple unsuccessful surgical interventions, along with a review of the relevant literature concerning the diagnosis and treatment of tailgut cysts. We chose to report this case to underscore the diagnostic challenges associated with retrorectal cystic hamartomas, particularly in cases where prior surgeries and chronic symptoms may confound clinical suspicion. The patient’s symptoms mimicked other more common anorectal or pelvic pathologies, leading to recurrent misdiagnoses and suboptimal surgical outcomes. Another key aspect of this case is the pivotal role of imaging tools, particularly MRI, in achieving the eventual diagnosis, especially when clinical and surgical findings fail to provide clarity.

## 2. Case Presentation

A 30-year-old woman was referred to our institution with a 3-year history of low abdominal pain. The patient had no other associated digestive symptoms, including rectal fullness, constipation, pain on defecation, rectal bleeding, or genitourinary obstruction symptoms. There were no abnormal findings at the general physical and per abdominal examination. The rectal examination was also normal. During her third pregnancy, a cystic pelvic mass had been diagnosed as a left ovarian cyst, with the suspicion of a dermoid origin. She underwent three consecutive surgical procedures, both laparoscopic and classical, for ovarian cysts, in different centers, without relieving symptoms. In all cases, the preoperative diagnosis was based on an abdominal and transvaginal ultrasound. At the third procedure, no ovarian cyst was found, and a computed tomography (CT) scan was recommended.

**Figure 1:**
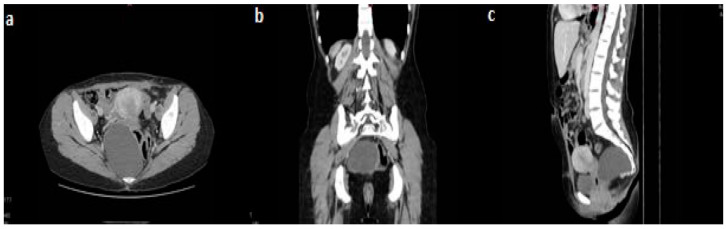
Contrast computed tomography showing the presacral tumor with right pararectal development; (**a**) axial view, arterial phase; (**b**) coronal view, arterial phase; (**c**) sagittal view, native phase.

The abdominal–pelvic CT scan revealed an 88/64 mm presacral tumor with a right pararectal development and a mass effect on the rectum, against which there was no cleavage plan; there was no relationship with the iliac vessels (Figure 1). A surgical resection of the lesion was decided upon, using an anterior approach via a Pfannenstiel incision. Intraoperatively, an 8 cm well-defined retroperitoneal presacral mass was described, which, during the handling maneuvers, tore up, eliminating an opaque, viscous liquid. A full resection of the cyst was performed. The postoperative recovery was uneventful, and the patient was discharged on the sixth postoperative day. One year after surgery, the patient had no evidence of recurrence, and was symptom-free.

**Figure 2:**
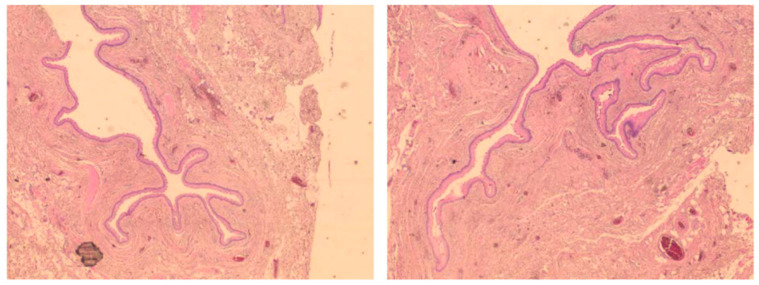
Overview of a large portion of the cyst wall (HE staining, ×40).

**Figure 3:**
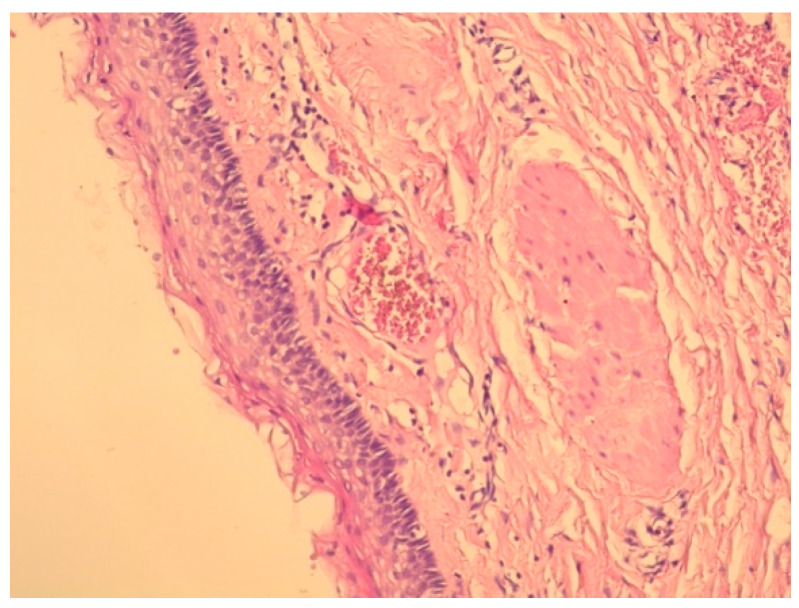
Cyst wall (detail): fibrovascular tissue with smooth muscle bundles lined by stratified squamous epithelium (HE staining, ×100).

**Figure 4:**
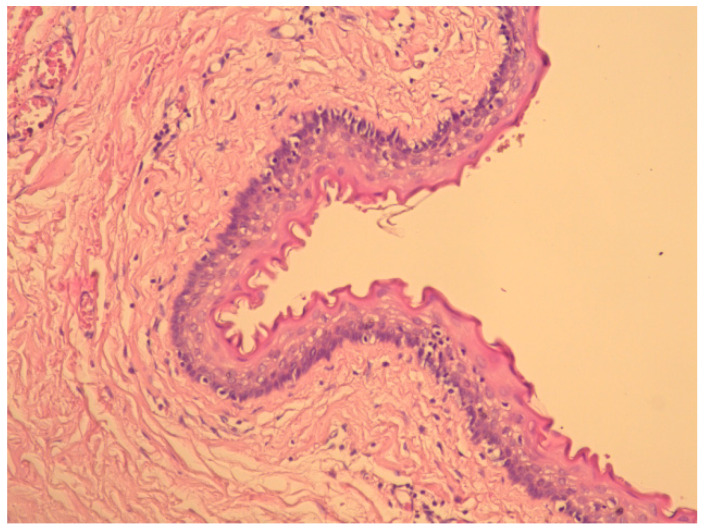
Cyst wall (detail) with rare inflammatory cells under the epithelium (HE staining, ×100).

Cytological examination of the fluid revealed a benign smear, and histopathology revealed an 8/3/1 cm cyst lined by non-keratinized stratified squamous epithelium without a granular layer, fibrous connective tissue walls with reduced inflammatory lymphoplasmacytic infiltration, and hemorrhage areas (Figure 2). Smooth muscle fibers were described at the periphery (Figure 3 and Figure 4). The final diagnosis was a retrorectal cystic hamartoma (tailgut cyst).

## 3. Materials and Methods

**Figure 5:**
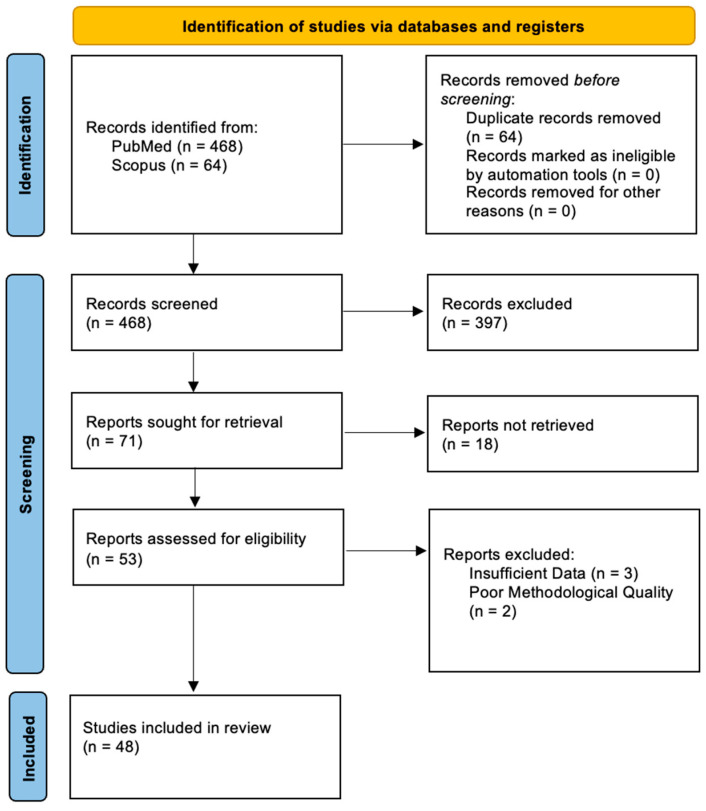
Flowchart depicting the search strategy employed to find the studies included in the review (following 2020 PRISMA guidelines).

This systematic review was conducted following the PRISMA 2020 guidelines. We conducted a comprehensive literature review on tailgut cysts, a rare congenital condition arising from remnants of the embryonic hindgut, focusing on including articles that present the clinical picture, diagnosis, management and outcomes of tailgut cysts. Our primary sources of data were PubMed and Scopus. Our terms included “tailgut cyst”, “retrorectal space” or “retrorectal hamartoma”. The inclusion criteria were articles published between January 1959 and October 2024, written in English, that provided full-text accessibility. The types of articles included original research, case reports, case series, systematic reviews or meta-analyses focusing on tailgut cysts. The exclusion criteria comprised duplicate articles or purely experimental manuscripts, articles published in languages other than English, or those that lacked sufficient details on clinical presentation, diagnosis management, or outcomes. The protocol followed is outlined in Figure 5.

## 4. Discussion

The tailgut cyst is a rare congenital anomaly typically located in retrorectal (presacral) space. This virtual anatomical space is bordered anteriorly by the posterior rectal wall, posteriorly by the sacrum, superiorly by the peritoneal reflection, and inferiorly by the levator ani and coccygeus muscles. Laterally, its boundaries include the ureters, iliac vessels, sacral nerve roots, and lateral rectal stalks. The rectosacral fascia further divides this space into two compartments, a superior and an inferior section. These compartments contain loose connective tissue, the middle sacral, iliolumbar, and middle hemorrhoidal vessels, along with branches of the sympathetic and parasympathetic nervous systems, as well as lymphatic structures [1,14,15].

A wide variety of masses can arise within retrorectal space. Differential diagnosis includes primary tumors (of neurogenic, osteogenic, or congenital origins), metastatic lesions, and inflammatory processes. Among congenital lesions, developmental cysts, chordomas, teratomas, and anterior sacral meningoceles are notable. Epidermoid and dermoid cysts share the presence of stratified squamous epithelium, but dermoid cysts also contain dermal components, such as sweat glands, hair follicles, and tooth buds [4,16].

The distinction between tailgut cysts and rectal duplication cysts lies in the cyst wall composition: tailgut cysts exhibit disorganized smooth muscle fibers without neural plexuses, whereas rectal duplication cysts have two layers of smooth muscle accompanied by nerve plexuses. Tailgut cysts can feature multiple epithelial types, sometimes within the same cyst, including stratified squamous, transitional, stratified columnar, mucinous or ciliated columnar, ciliated pseudostratified columnar, and gastric epithelium. They are typically uni- or multilocular with thin walls, and their contents range from clear fluid to dense mucus [16,17,18].

The prevailing hypothesis for the development of tailgut cysts suggests that these lesions originate from vestigial remnants of the embryonic hindgut. During early embryogenesis, the embryo possesses a true tail until approximately the 35th day of gestation, extending caudally beyond the future site of the anus. This primitive hindgut extends into the embryonic tail, giving rise to the terms “postanal gut” and “tailgut.” Normally, this structure undergoes complete involution by the 56th day of gestation. However, incomplete involution may result in the persistence of tailgut remnants, which are hypothesized to contribute to the formation of tailgut cysts [5,17,19].

The estimated incidence of this condition is 1 in 40,000 individuals. While cases have been reported in neonates, tailgut cysts predominantly affect women in the third to sixth decades of life, with a female-to-male ratio of 3:1. No specific risk factors have been identified in the literature [14,20,21,22,23].

The clinical presentation of tailgut cysts is often nonspecific, leading to delayed or incidental diagnoses. According to a study by Singer et al., an average of 4.7 invasive procedures or surgeries is typically required to achieve an accurate diagnosis and appropriate treatment of a retrorectal mass [17]. Many patients remain asymptomatic for extended periods, with symptoms usually emerging only when complications occur. These complications include infection, fistula formation, bleeding, or malignant transformation, with infection being the most common (40–50%). Rectal bleeding is more characteristic of rectal duplication cysts [10,24,25,26].

Symptoms typically arise due to a mass effect on adjacent structures, and may include lower abdominal pain, back pain, rectal fullness, constipation, painful defecation, altered stool patterns, genitourinary obstruction, dysuria, pollakiuria, or right-sided sciatica [1,3,6,13]. In rare cases, it is found during labor, where a tailgut cyst may obstruct the birth canal, necessitating a transition to cesarean delivery [14]. On digital rectal examination, these lesions are often identified as extrinsic masses with a fluctuant consistency, further aiding in clinical suspicion [14].

Advancements in imaging techniques have significantly improved the diagnostic accuracy for this rare lesion. A transrectal ultrasound (TRUS) is particularly useful in patients presenting with rectal bleeding, providing valuable information regarding the location, size, content, and potential local invasion of the lesion, while also aiding in the exclusion of other differential diagnoses [6,18]. Computed tomography (CT) characterizes tailgut cysts as well-circumscribed, uni- or multilocular masses with liquid or soft-tissue density. In cases of infection, diffuse wall thickening may be evident. Features such as intra-cystic proliferation, septations, irregular or nodular thickening, involvement of adjacent structures, or lymphadenopathy on CT may raise suspicion for malignant transformation [18]. The preferred imaging modality for retrorectal masses is magnetic resonance imaging (MRI), which offers superior anatomical detail, excellent soft tissue contrast, and the advantage of multiplanar reconstruction [1,14,17]. On MRI, retrorectal tumors typically exhibit low signal intensity on T1-weighted images and high signal intensity on T2-weighted images, although these characteristics can vary with the composition of the cyst. For instance, increased protein concentration (e.g., from bleeding) can produce hyperintense signals on T1-weighted images; mucinous fluid, depending on its protein content and viscosity, may appear hypointense to hyperintense on both T1- and T2-weighted images. Malignant degeneration is suggested by a consistently low signal intensity on both T1- and T2-weighted images, while septations generally appear hypointense on T2-weighted images [14,27,28]. Aflalo-Hazan et al. observed that most tailgut cysts demonstrate high signal intensity relative to muscle tissue on MRI [29]. Kim et al. further described a characteristic MRI finding of tailgut cysts as multilocular masses with internal septations on T2-weighted images [30]. These imaging features aid in distinguishing tailgut cysts from other retrorectal lesions.

A controversy arises regarding the necessity of a preoperative biopsy. Most physicians agree that the disadvantages outweigh the advantages, this procedure being usually discouraged [31,32,33,34]. The risks that may occur include biopsy tract or peritoneal dissemination, bleeding, or infection. Some authors support the necessity of a preoperative biopsy for malignant suspected lesions, evoking the possibility of neoadjuvant therapy for certain pathological subtypes before surgery, which may improve the final result [14,17]. Hjermstad et al. presented a 2% rate of malignancy in a series of 53 tailgut cysts, but it is assumed that this rate is much higher, with Mayo Clinic Group reporting a 13% rate in a paper published in 2010 [35,36]. The most common malignant degenerations of tailgut cysts are adenocarcinomas, carcinoids, and sarcomas [17,18].

Complete surgical resection is the treatment of choice for tailgut cysts [1,18]. Over the past few years, different approaches have been described in the literature: the anterior approach (transabdominal), the posterior approaches, and a combination of these two. The posterior approaches include many surgical techniques, like inter-sphincteric, trans-sphincteric, para-sacrococcygeal, trans-sacral, trans-sacrococcygeal, trans-anorectal, or trans-vaginal. The transabdominal approach offers a direct image of pelvic structures, the ureters and iliac vessels. The advantage of the posterior approach is the access to the distal component of the tumor, but the lack of control over pelvic structures and the possibility of injuring the pelvic nerves is higher [1,10]. The surgical technique selection is usually based on several factors, such as the size, location, and morphology of the lesion, and possible adherences, infection or malignancy. It is thought that the anterior approach should be carried out for lesions that are above S3 or the sacral promontory, and the posterior approach should be reserved for masses that are below the above mentioned point [1,17,34]. For the posterior approaches the patient should be placed in a jack knife, lithotomy or lateral position and all the efforts be made to not injure and to preserve the sacral nerve roots. If this is not an option, the bladder and the bowel are thought to keep a normal function even with unilateral preservation of S2–S4 nerve roots. The posterior approach may be a good choice if the proximal extent of the mass can be palpated at the digital rectal examination. The combined method sums up the advantages of the two approaches, offering a proper view of the anatomy, the lesion’s extent, and the capacity of a better vascular control, being used for large masses that are located above and below the S3 vertebrae or tailgut cysts with a diameter greater than 4–5 cm [13]. Laparoscopic excision was also per formed by some authors with promising results [37]. Transanal endoscopic microsurgery excisions may also become a feasible option [38]. The advantages of these modern techniques include a good exposure of the presacral space, a reduced surgical trauma and an easier recovery [39]. The disadvantages are represented by the high costs, being carried out only in centers of expertise. The patient must be well informed about the limitations and the risks of surgery, explaining the possibility of a stoma if necessary [1,14,34]. Although surgical excision represents the gold standard treatment for tailgut cysts, nonsurgical management should be taken into account in small tailgut cysts, asymptomatic patients in order to postpone the surgery, high-risk patients who are likely to experience significant postoperative complications, or those who prefer less invasive procedures [40,41,42,43]. An alternative would be fine-needle aspiration guided by endosonography or CT-guided drainage of the cysts, with or without alcohol injection. These procedures must be carefully performed, and follow-up is mandatory, due to the risks they carry, such as infection, recurrence, or malignant dissemination [41,42,44,45,46]. Nonetheless, nonsurgical management may be appropriate in carefully selected cases, emphasizing the importance of individualized treatment strategies.

Postoperatively, long-term complications could appear, which includes pelvic floor dysfunction, delayed wound healing, sexual dysfunction, and a risk of recurrence that may reach 16% [20,28,47,48]. There has not been established a standard follow-up, but an annual digital exam and a CT scan in the first and the fifth year after surgery should be performed [13].

## 5. Conclusions

This report emphasizes the challenges associated with retrorectal masses and underscores the importance of considering tailgut cysts as a differential diagnosis in cases of perirectal tumors, particularly in patients with a history of multiple failed procedures and surgeries. MRI is the most important investigation used preoperatively when the suspicion of retrorectal cystic hamartomas arises. The cornerstone of these uncommon lesions is surgical excision, with less invasive and promising techniques in the future.

## Data Availability

Data are contained within the article—The original contributions presented in this study are included in the article. Further inquiries can be directed to the corresponding author(s).

## References

[1] Kildušis E., Samalavičius N.E. (2014). Surgical management of a retro-rectal cystic hamartoma (tailgut cyst) using a trans-rectal approach: A case report and review of the literature. J. Med. Case Rep..

[2] Bulisani B.M., Gomes L.G.L., Leite M.A.d.O., Moreno R., Rodrigues M.R., Rossi F.M.B., da Silva R.B.F., do Carmo L.C.B., Waisberg J. (2023). Robotic approach to remove four tailgut cyst cases in Brazil: A case series. Einstein.

[3] Haval S., Dwivedi D., Nichkaode P. (2024). Presacral Tailgut Cyst. Ann. Afr. Med..

[4] Wang G., Miao C. (2023). Chinese expert consensus on standardized treatment for presacral cysts. Gastroenterol. Rep..

[5] Malliou P., Syrnioti A., Koletsa T., Karlafti E., Karakatsanis A., Raptou G., Apostolidis S., Michalopoulos A., Paramythiotis D. (2022). Mucinous adenocarcinoma arising from a tailgut cyst: A case report. World J. Clin. Oncol..

[6] Soltany S. (2016). A Rare Coexistence of Retrorectal and Ovarian Cysts: A Case Report. Iran. Red Crescent Med. J..

[7] Karn S., Huda F., David L.E., Sharma J., Prajapati T., Chauhan U., Singh S.K., Basu S. (2022). Recurrent retrorectal tailgut cyst mimicking deep pelvic abscess: A diagnostic dilemma. Radiol. Case Rep..

[8] El Yamine O., Fatine A., Boufettal R., Errguibi D., Hajri A., El Jay S.R., Chehab F. (2021). Retrorectal cystic hamartoma: A case report. Ann. Med. Surg..

[9] Elkarouachi A., Assemar M., El Jai S.R.J., Erguibi D., Boufettal R., Chehab F. (2021). Retrorectal cystic hamartoma: A case report. Int. J. Surg. Case Rep..

[10] Naseri A., Behboudi B., Faryabi A., Tafti S.M.A., Sharifi A., Keramati M.R., Fazeli M.S., Keshvari A., Zeinalizadeh M., Asbagh R.A. (2022). Surgical management of retrorectal tumors: A single-center 12 years’ experience. Ann. Coloproctol..

[11] La Greca G., Trombatore G., Basile G., Conti P. (2020). Retrorectal tumors: Case report and review of literature. Int. J. Surg. Case Rep..

[12] Sakr A., Kim H.S., Han Y.D., Cho M.S., Hur H., Min B.S., Lee K.Y., Kim N.K. (2019). Single-center Experience of 24 Cases of Tailgut Cyst. Ann. Coloproctol..

[13] Haydar M., Griepentrog K. (2015). Tailgut cyst: A case report and literature review. Int. J. Surg. Case Rep..

[14] Joyce E.A., Kavanagh D.O., Winter D.C. (2012). A rare cause of low back pain: Report of a tailgut cyst. Case Rep. Med..

[15] George M., Alina-Roxani G., Zoi T., Maria O., George C., Charalampos C. (2018). A Rare Case Report of a Tail-Gut Cyst from a Gynecological Point of View. J. Family Reprod. Health.

[16] Suhani Meena K., Ali S., Aggarwal L., Thomas S. (2014). Retrorectal cystic hamartoma: A problematic “tail”. J. Surg. Tech. Case Rep..

[17] Bathla L., Singh L., Agarwal P.N. (2013). Retrorectal cystic hamartoma (tailgut cyst): Report of a case and review of literature. Indian J. Surg..

[18] Rosa G., Lolli P., Vergine M., El-Dalati G., Malleo G. (2012). Surgical excision of developmental retrorectal cysts: Results with long-term follow-up from a single institution. Updates Surg..

[19] Liang F., Li J., Yu K., Zhang K., Liu T., Li J. (2020). Tailgut Cysts with Malignant Transformation: Features, Diagnosis, and Treatment. Med. Sci. Monit..

[20] Jehangir A., Le B.H., Carter F.M. (2016). A rare case of carcinoid tumor in a tailgut cyst. J. Community Hosp. Intern. Med. Perspect..

[21] Azatcam M., Altun E., Avci V. (2018). Histopathological diagnostic dilemma in retrorectal developmental cysts: Report of a case and review of the literature. Turk. J. Pathol..

[22] Chung K.Y., Lee N.M., Choi E.S., Yoo B.H., Kim G.J., Cha S.J., Kim G.H., Kim M.K. (2013). A tailgut cyst-cystic mass diagnosed by prenatal ultrasonography. Am. J. Perinatol. Rep..

[23] Raisolsadat S.M.A., Zabolinejad N., Tabrizian-Namini F., Faraji P. (2013). Tailgut cyst in an infant with imperforate anus: A case report. Iran. J. Pediatr..

[24] Saba L., Fellini F., Greco F.G., Leonzio A., Cionci G., Consolo D., Ariozzi I., Zambrini E.I., Bocchi C., Concari G. (2014). MRI evaluation of not complicated Tailgut cyst: Case report. Int. J. Surg. Case Rep..

[25] Rompen I.F., Scheiwiller A., Winiger A., Metzger J., Gass J.M. (2021). Robotic-Assisted Laparoscopic Resection of Tailgut Cysts. J. Soc. Laparosc. Robot. Surg..

[26] Fang Y., Zhu Y., Liu W.Z., Zhang X.Q., Zhang Y., Wang K. (2022). Malignant transformation of perianal tailgut cyst: A case report. World J. Gastrointest. Surg..

[27] Peter P., George U., Peacock M. (2010). Retrorectal hamartoma: A “tail” of two cysts!. Indian J. Radiol. Imaging.

[28] Shetty A.S., Loch R., Yoo N., Mellnick V., Fowler K., Narra V. (2015). Imaging of tailgut cysts. Abdom. Imaging.

[29] Aflalo-Hazan V., Rousset P., Mourra N., Lewin M., Azizi L., Hoeffel C. (2008). Tailgut cysts: MRI findings. Eur. Radiol..

[30] Kim J., Jin S.Y., Hong S., Lee T. (2014). A carcinoid tumour arising within a tailgut cyst: A diagnostic challenge. Scott. Med. J..

[31] Fechner K., Bittorf B., Langheinrich M., Weber K., Brunner M., Grützmann R., Matzel K.E. (2024). The management of retrorectal tumors—A single-center analysis of 21 cases and overview of the literature. Langenbecks Arch. Surg..

[32] Wang Y.S., Guo Q.Y., Zheng F.H., Huang Z.W., Yan J.L., Fan F.X., Liu T., Ji S.X., Zhao X.F., Zheng Y.X. (2022). Retrorectal mucinous adenocarcinoma arising from a tailgut cyst: A case report and review of literature. World J. Gastrointest. Surg..

[33] Poskus E., Makunaite G., Kubiliute I., Danys D. (2018). Case report: Laparoscopic approach in the treatment of presacral lipoma. Ann. Med. Surg..

[34] Grossi U., Santoro G.A., Sarcognato S., Iacomino A., Tomassi M., Zanus G. (2021). Perianal Tailgut Cyst. J. Gastrointest. Surg..

[35] Hjermstad B.M., Helwig E.B. (1988). Tailgut cysts. Report of 53 cases. Am. J. Clin. Pathol..

[36] Mathis K.L., Dozois E.J., Grewal M.S., Metzger P., Larson D.W., Devine R.M. (2010). Malignant risk and surgical outcomes of presacral tailgut cysts. Br. J. Surg..

[37] Kashima H., Teraishi F., Matsumi Y., Shimamura H., Fujiwara T. (2024). Laparoscopic Resection Combined with a Transsacral Approach for a Recurrent Tailgut Cyst with a Refractory Fistula. Acta Medica Okayama.

[38] Bardol T., Souche R., Druet C., Bertrand M.M., Ferrandis C., Prudhomme M., Borie F., Fabre J.M. (2024). Minimally invasive approach for retrorectal tumors above and below S3: A multicentric tertiary center retrospective study (MiaRT study). Tech. Coloproctol..

[39] Kwak H.D., Ju J.K. (2020). Laparoscopic Resection of a Huge Retrorectal Tumor. Ann. Coloproctol..

[40] Hopper L., Eglinton T.W., Wakeman C., Dobbs B.R., Dixon L., Frizelle F.A. (2016). Progress in the management of retrorectal tumours. Color. Dis..

[41] Aljuhani F., Almunami B., Alsamahi R., Malibary N., Algaithy Z. (2019). Alcohol injection for nonsurgical management of tailgut cyst in a middle-aged woman: A case report. Clin. Case Rep..

[42] Killingsworth C., Gadacz T.R. (2005). Tailgut cyst (retrorectal cystic hamartoma): Report of a case and review of the literature. Am. Surg..

[43] Hall D.A., Pu R.T., Pang Y. (2007). Diagnosis of foregut and tailgut cysts by endosonographically guided fine-needle aspiration. Diagn. Cytopathol..

[44] Rathinamanickam H., Pawa S. (2015). A Tailgut Cyst Diagnosed by Endoscopic Ultrasound-Guided Fine-Needle Aspiration. ACG Case Rep. J..

[45] Gönül I.I., Bağlan T., Pala I., Menteş B. (2007). Tailgut cysts: Diagnostic challenge for both pathologists and clinicians. Int. J. Color. Dis..

[46] Prasad A.R., Amin M.B., Randolph T.L., Lee C.S., Ma C.K. (2000). Retrorectal cystic hamartoma: Report of 5 cases with malignancy arising in 2. Arch. Pathol. Lab. Med..

[47] Burke J.R., Shetty K., Thomas O., Kowal M., Quyn A., Sagar P. (2022). The management of retrorectal tumours: Tertiary centre retrospective study. BJS Open.

[48] Aubert M., Mege D., Parc Y., Rullier E., Cotte E., Meurette G., Zerbib P., Trilling B., Lelong B., Sabbagh C. (2021). Surgical Management of Retrorectal Tumors: A French Multicentric Experience of 270 Consecutives Cases. Ann. Surg..

